# CD36 gene polymorphism rs1761667 (G > A) is associated with hypertension and coronary artery disease in an Iranian population

**DOI:** 10.1186/s12872-019-1111-6

**Published:** 2019-06-11

**Authors:** Mohammad Amin Momeni-Moghaddam, Gholamreza Asadikaram, Hamed Akbari, Moslem Abolhassani, Mohammad Masoumi, Zahra Nadimy, Mohammad Khaksari

**Affiliations:** 1 Neuroscience Research Center, Institute of Neuropharmacology, Kerman University of Medical Sciences, Kerman, Iran; 2 Department of Biochemistry, School of Medicine, Kerman University of Medical Sciences, Kerman, Iran; 3 Endocrinology and Metabolism Research Center, Institute of Basic and Clinical Physiology Sciences, Kerman, Iran; 4 Cardiovascular Research Center, Institute of Basic and Clinical Physiology Sciences, Kerman University of Medical Sciences, Kerman, Iran; 5 Physiology Research Center, Institute of Neuropharmacology, Kerman University of Medical Sciences, Kerman, Iran

**Keywords:** CD36, Coronary artery disease, Hypertension, Polymorphism, SNP

## Abstract

**Background:**

CD36 is associated with regulation of lipid metabolism, atherosclerosis, and blood pressure. Moreover, its variation may be involved in the development of hypertension and/or coronary artery disease (CAD). The present study was conducted to investigate the possible association of CD36 rs1761667 (G > A) polymorphism with hypertension and/or CAD in the southeastern of Iran.

**Methods:**

The present observational study was composed of 238 subjects who were admitted for coronary angiography, and divided into four groups: 1) hypertensive without CAD (H-Tens, *n* = 52); 2) hypertensive with CAD (CAD + H-Tens, *n* = 57); 3) CAD without hypertension (CAD, *n* = 65); and 4) non-hypertensive without CAD as the control group (Ctrl, *n* = 64). The CD36 rs1761667 polymorphism was genotyped with PCR-RFLP method. Association between CD36 rs1761667 genotypes and the risk of CAD and hypertension was assessed using multinomial regression by adjusting for age, sex, creatinine, fasting blood sugar (FBS), systolic blood pressure (SBP) and diastolic blood pressure (DBP).

**Results:**

In the present study, minor allele (A) frequency was 0.36. The genotype, but not allele frequency of the CD36 rs1761667 was significantly different between the four study groups (*p* = 0.003). Furthermore, using a recessive inheritance model CD36 rs1761667 polymorphism was significantly associated with an increased risk of CAD with hypertension (OR = 5.677; 95% CI = 1.053–30.601; *p* = 0.043). However, using the dominant model of CD36 rs1761667 had a protective effect on H-Tens and CAD patients.

**Conclusion:**

The present findings revealed an association between CD36 rs1761667 polymorphism and susceptibility to hypertension and/or CAD in a southeastern Iranian population.

## Background

Cardiovascular disease (CVD) is one of the most important threats to human life around the world, and includes sub-categories such as coronary artery disease (CAD), acute coronary syndrome (ACS) and ischemic cardiomyopathy [1]. CAD is a multifactorial disorder which develops and progresses as a result of both environmental and genetic factors [2]. Various risk factors are involved in the development of CAD, including, atherosclerosis, hypertension, smoking, lifestyle, high fat diet, non-exercise and diabetes mellitus (DM) [1, 3–7].

CD36, a transmembrane glycoprotein, is a member of class B scavenger receptor family. In addition to monocytes and macrophages, it is expressed in a number of other cells, such as adipocytes, endothelial cells, and platelets. Multiple ligands are attached to this receptor and CD36 participates in many biological processes [8].

Recently, various studies have revealed results about the relationship between CD36 gene polymorphisms and obesity on the one hand and CD36 gene polymorphisms, hypertension, and CVDs on the other. The CD36 gene with 32 kb is located on the chromosome 7 (q11.2) and contains 15 exons [9]. SNP rs1761667 (G > A) is located in intron of 5′ flanking exon 1A [10]. The CD36 receptor has high-affinity for long chain fatty acids (FAs) and is involved in metabolism of lipids. The A allele of rs1761667 decreases the CD36 expression and is related with upper recognition taste thresholds for fat (i.e., lower oral sensitivity to fat) [11]. Moreover, clinical investigations have shown that polymorphisms of this gene (such as rs1761667, rs10499859, rs3173798 and rs1049673) can influence the lipid metabolism, cardiovascular risk, essential hypertension, insulin resistance, familial type 2 diabetes mellitus (T2DM) and body mass index (BMI) [12–14]. For example, this receptor has high-affinity for long chain fatty acids (FAs) and is involved in lipid metabolism.

It is believed that CD36 polymorphisms confer susceptibility for hypertension and/or CAD due to the roles of CD36 in the regulation of lipid metabolism, atherosclerosis, and blood pressure [12, 15, 16].

Therefore, the present study was conducted aiming to evaluate the CD36 rs1761667 polymorphism in patients with CAD and hypertension in a southeastern Iranian population.

## Methods

### Study subjects

**Fig. 1:**
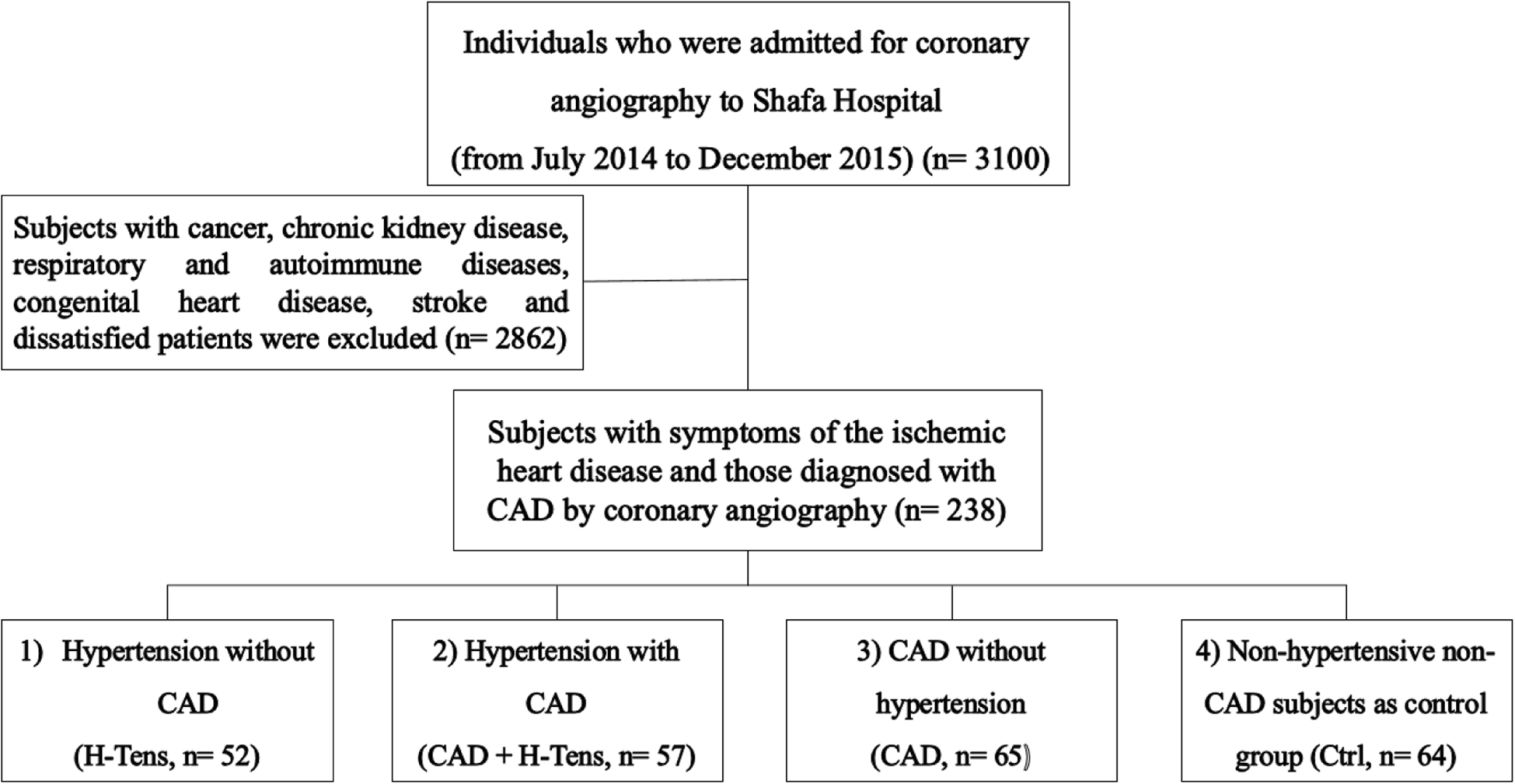
Selection of study population

This observational study was performed on a total of 238 individuals who were admitted for coronary angiography to Shafa Hospital of Kerman University of Medical Sciences, Kerman, Iran (from July 2014 to December 2015). Subject selection process was conducted as described elsewhere [17–19]. Briefly, all CAD-suspected participants were examined by coronary angiography, before which they went through CAD treatment as they were symptomatic, had a history of hospitalization in coronary care unit (CCU), or there was evidence of myocardial ischemia in noninvasive investigations such as an exercise test or perfusion imaging. The inclusion criteria were as follows: participants’ consent, subjects with symptoms of the ischemic heart disease and those diagnosed with CAD by coronary angiography. Subjects suffering from cancer, chronic kidney, respiratory and autoimmune diseases, congenital heart disease, and stroke were excluded [17]. The subjects were divided into four groups: 1) hypertensive without CAD (H-Tens, *n* = 52); 2) hypertensive with CAD (CAD + H-Tens, *n* = 57); 3) CAD without hypertension (CAD, *n* = 65); and 4) non-hypertensive without CAD as the control group (Ctrl, *n* = 64) (Fig. 1).

This study conforms to the *Declaration of Helsinki* regarding research involving human subjects and was approved by the Ethics Committee of Kerman University of Medical Sciences, Kerman, Iran (IR.KMU.REC.1395.261).

### Samples and data collection

Interviews were conducted to gather demographic data from all participants, each of whom provided an approximate amount of 10 ml of blood, of which 5 ml was collected into vacutainer tubes containing EDTA (for DNA extraction) and 5 ml into vacutainer tubes without anti-coagulation agent. The samples were stored at room temperature and then centrifuged for serum separation. The serum samples were aliquoted and frozen at − 20 °C to be used for subsequent experiments. Fasting blood sugar (FBS), total cholesterol (TC), triglycerides (TG), high-density lipoprotein cholesterol (HDL-C), low-density lipoprotein cholesterol (LDL-C), sodium (Na), potassium (K), creatinine (Cr), and urea were determined using commercially available kits (Pars Azmoon, Tehran, Iran). BMI was calculated as weight divided by the square of height (kg/m^2^). Blood pressure was also measured from the right arm using an automatic sphygmomanometer during supine rest with 10-min intervals, and an average number of three blood pressure measurements were used. The criteria for occurrence of hypertension were as follow: a systolic blood pressure (SBP) ≥ 140 mmHg, a diastolic blood pressure (DBP) ≥ 90 mmHg and receiving antihypertensive medication.

### Genotyping

Genomic DNA was extracted from white blood cells using the salting out method [20].

The concentration of extracted DNA was measured using Nanodrop spectrophotometer (ND-1000, ThermoFisher Scientific, Wilmington, USA). It was then frozen at − 70 °C to be used for subsequent experiments. Genotypes of CD36-rs1761667 polymorphism were determined using the polymerase chain reaction-restriction fragment length polymorphism (PCR-RFLP) method as described below.

The SNP was detected by PCR using following primers: 5′- CAAGGTCTGGTATCCACCTGTT − 3′ (forward), 5′- ATGAAGCTTCCCGCCTTAGAA − 3′ (reverse) and amplification was carried out using a Biometra T advanced (Analytik Jena, Germany). PCR conditions were as follows: initial denaturation at 95 °C for 5 min, followed by 35 cycles of amplification including denaturation at 95 °C, annealing at 60 °C, extension at 72 °C (each comprising 30 s), and the final extension at 72 °C for 5 min. PCR products (10 μl) were digested with *HhaI* restriction endonuclease (Thermo Scientific, USA) at 37 °C for 16 h and fragments were separated by agarose gel electrophoresis (1.5% agarose) stained with ethidium bromide. Afterwards, being observed by ultraviolet light (UV Tec Cambridge Gel doc), three genotypes of CD36-rs1761667 were detected which include: GG (161 and 264 bp), GA (161, 245, and 425 bp), and AA (425 bp).

### Statistical analysis

All continuous and categorical variables were expressed, respectively, in the form of mean ± standard deviation (M ± SD) and counts (percentages). The differences among groups were assessed using one-way analysis of variance (ANOVA)/Kruskal-Wallis with post-hoc Tukey/Mann-Whitney U-tests as well as Chi-square/Fisher’s exact tests. Deviation from the Hardy-Weinberg equilibrium (HWE) was tested by the Chi-square test. To examine the independent roles that the genotypes play against hypertension and CAD risk, multinomial logistic regression was carried out. The statistical analyses were performed using SPSS software version 23.0 for Windows (SPSS Inc., Chicago, IL). The *p*-values less than 0.05 were considered to be statistically significant. Raw *p*-values are reported and no correction for multiple testing was applied. Power analysis was performed using the Power and Sample Size Calculation G* Power software (version 3.1.9.2). In the post hoc power analysis, 1-beta (power) computed as a function of alpha (*p*-value) and the sample size(s) used in the study. The significant level was considered as 0.05.

## Results

### Demographics analysis

Demographic and biochemical parameters of all participants are presented in Table 1. There were significant differences in sex, Cr, SBP and DBP between the four groups (*p < 0.001, p = 0.012, p = 0.002,* and *p = 0.017, respectively*). However, with regard to other factors, no significant difference was found between the four study groups. In order to eliminate the effects of confounders such as age and sex, as well as adjusting for the baseline values of the desired variables (FBS, Cr, blood pressure, and etc.), these variables were included in regression model.

**Table 1:** Demographics and biochemical parameters of subjects among four groups

Variables	H-Tens (*N* = 52)	CAD + H-Tens (*N* = 57)	CAD (*N* = 65)	Ctrl (*N* = 64)	*P*-value
Age, years	54.69 ± 9.05	56.52 ± 11.00	56.20 ± 9.78	54.00 ± 10.40	0.424
Sex (m/f)	27/25	44/13	54/11	39/26	< 0.001^b^
BMI (kg/m^2^)	27.02 ± 5.48	24.92 ± 4.48	24.52 ± 4.37	25.08 ± 5.12	0.057^c^
FBS (mg/dl)	94.23 ± 12.68	95.30 ± 18.95	94.30 ± 15.13	95.56 ± 14.58	0.860^c^
TG (mg/dl)	116.35 ± 58.33	109.57 ± 56.25	111.90 ± 58.06	107.92 ± 37.93	0.788^c^
TC (mg/dl)	143.13 ± 32.82	136.38 ± 34.17	144.03 ± 38.52	149.05 ± 45.97	0.492^c^
HDL-c (mg/dl)	38.21 ± 9.72	37.11 ± 9.62	38.92 ± 10.70	41.63 ± 10.32	0.061^a^
LDL-c (mg/dl)	79.84 ± 30.91	77.46 ± 27.65	83.01 ± 31.80	83.43 ± 41.38	0.938^c^
Na (mmol/l)	139.60 ± 3.23	139.27 ± 3.13	139.70 ± 2.95	139.18 ± 2.99	0.702^c^
K (mmol/l)	4.34 ± 0.44	4.20 ± 0.36	4.34 ± 0.43	4.31 ± 0.44	0.311^c^
Cr (mg/dl)	0.98 ± 0.22	1.07 ± 0.20	1.10 ± 0.19	1.04 ± 0.23	0.012^c^
Urea (mg/dl)	35.48 ± 10.36	35.39 ± 9.10	36.28 ± 10.24	34.76 ± 12.67	0.753^c^
DBP (mmHg)	74.54 ± 9.32	72.73 ± 12.06	70.08 ± 8.03	75.73 ± 8.28	0.017^c^
SBP (mmHg)	123.02 ± 14.58	121.25 ± 13.25	115.33 ± 11.61	122.14 ± 14.21	0.002^c^

*H-Tens* Hypertension, *CAD + H-Tens* Coronary Artery Disease and Hypertension, *CAD* Coronary Artery Disease, *Ctrl* Control; *BMI* Body mass index, *FBS* Fasting blood sugar, *TC* Total cholesterol, *TG* Triglycerides, *HDL-c* High-density lipoprotein cholesterol, *LDL* Low-density lipoprotein cholesterol, *Na* Sodium, *K* potassium, *Cr* Creatinine, *SBP* Systolic Blood Pressure, *DBP* Diastolic Blood Pressure. Continuous and categorical values are presented as mean ± SD and number, respectively

^a^ANOVA followed by post-hoc Tukey test

^b^*chi*-square test

^c^Kruskal-Wallis followed by post-hoc Mann-Whitney U-test

### Genotype and allele frequency distribution of CD36 rs1761667

Genotype and allele frequency distributions of CD36 rs1761667 among the study groups are provided in Table 2. At the CD36 rs1761667, significant difference was found in genotype frequency between the four groups (*p* = 0.003). Additionally, regarding the genotype frequency of CD36 rs1761667, significant differences were observed between H-Tens and Ctrl groups (*p* = 0.001). Likewise, the CAD + H-Tens group showed a significant difference in the genotype distribution compared with H-Tens patients and Ctrl groups (*p* = 0.029 and *p* = 0.034, respectively). Moreover, H-Tens patients were shown to have a significantly lower A allele frequency compared to CAD + H-Tens and Ctrl subjects (*p* = 0.050 and *p* = 0.045, respectively). However, there were no significant differences regarding alleles between the other groups (*p*>0.05 for all comparisons). The genotypic frequencies in control group were in accordance with HWE and other groups were not in equilibrium (*P =* 0.005) (Table 2).

**Table 2:** Genotype and allele frequencies of CD36 rs1761667 in the four study groups

SNP		(1) H-Tens (*N* = 52)	(2) CAD + H-Tens (*N* = 57)	(3) CAD (*N* = 65)	(4) Ctrl (*N* = 64)	*P*-value						
						1 vs. 2	1 vs. 3	1 vs. 4	2 vs. 3	2 vs. 4	3 vs. 4	*1&2&3&4* ^*€*^
rs1761667												
GG		6 (0.12)	17 (0.30)	12 (0.18)	26 (0.4)	0.029	0.563	0.001	0.103	0.034	0.016	0.003
GA		40 (0.77)	38 (0.67)	45 (0.7)	30 (0.46)							
AA		6 (0.12)	2 (0.03)	8 (0.12)	9 (0.14)							
Allele G		52 (0.5)	72 (0.63)	69 (0.53)	82 (0.63)	0.050	0.640	0.045	0.112	0.990	0.102	0.086
Allele A		52(0.5)	42 (0.37)	61 (0.47)	48 (0.37)							
HWE	*P*-value	0.0001	0.001	0.001	0.941							
	X^2^	15.076	10.664	9.880	0.005							

(1) *H-Tens* Hypertension, (2) *CAD + H-Tens* Coronary Artery Disease and Hypertension, (3) *CAD* Coronary Artery Disease, (4) *Ctrl* Control, *HWE* Hardy-Weinberg Equilibrium. Data are presented as number (%).The differences of CD36 rs 1,761,667 genotype and allele frequencies between study groups were analyzed using Chi-square/Fisher’s exact tests

^€^significant difference between the four groups (*p* < 0.05)

Table 3 depicts the comparison of demographic and laboratory parameters between the CD36 rs1761667 genotype groups among H-Tens, CAD + H-Tens, CAD and Ctrl individuals. CAD + H-Tens patients with GG (145.06 ± 80.98) genotypes showed significantly higher levels of TG compared to both GA (94.24 ± 32.39) and AA (91.50 ± 24.74) genotypes (*p* = 0.018). Furthermore, significantly lower levels of Na and K were found in GG genotype of CD36 rs1761667 between CAD patients as compared to GA (*p* = 0.029 and *p* = 0.002, respectively). However, regarding demographic and laboratory factors, no significant differences were observed between CD36 rs1761667 genotypes in Ctrl subjects (*p*>0.05 for all comparisons).

**Table 3:** Patient characteristics and distribution of CD36 rs1761667 genotypes in H-Tens, CAD + H-Tens, CAD and Ctrl subjects

		Age (years)	BMI (kg/m^2^)	FBS (mg/dl)	TG (mg/dl)	TC (mg/dl)	HDL-c (mg/dl)	LDL-c (mg/dl)	Na (mmol/l)	K (mmol/l)	Cr (mg/dl)	Urea (mg/dl)	SBP (mmHg)	DBP (mmHg)
H-Tens	GG	55.50 ± 6.95	27.35 ± 9.43	97.17 ± 6.55	90.33 ± 22.59	144.17 ± 24.28	43.67 ± 5.57	82.43 ± 26.70	140.33 ± 4.32	4.43 ± 0.33	0.89 ± 0.16	34.50 ± 5.28	116.67 ± 10.32	71.67 ± 7.52
	GA	54.90 ± 9.86	27.11 ± 5.05	94.90 ± 11.42	118.68 ± 63.43	140.55 ± 32.75	37.95 ± 9.65	75.89 ± 28.86	139.33 ± 3.11	4.34 ± 0.42	0.99 ± 0.17	35.88 ± 10.72	124.43 ± 15.31	74.78 ± 9.01
	AA	52.50 ± 4.68	26.09 ± 4.20	86.83 ± 22.40	126.83 ± 42.75	159.33 ± 40.80	34.50 ± 12.39	103.63 ± 41.48	140.67 ± 3.14	4.30 ± 0.70	0.95 ± 0.46	33.83 ± 12.84	120.00 ± 12.64	75.83 ± 13.57
	*P*-value	0.816	0.906	0.825	0.323	0.433	0.252	0.119	0.545	0.869	0.483	0.682	0.519	0.712
	GG	57.47 ± 12.84	25.37 ± 3.39	93.76 ± 16.23	145.06 ± 80.98	145.18 ± 35.81	34.18 ± 6.53	81.98 ± 27.67	139.24 ± 3.23	4.31 ± 0.32	1.08 ± 0.21	35.59 ± 10.13	122.06 ± 13.17	69.88 ± 17.39
CAD + H-Tens	GA	56.43 ± 10.21	24.75 ± 4.92	96.43 ± 20.60	94.24 ± 32.39^#^	131.11 ± 32.67	37.73 ± 9.96	74.69 ± 27.83	139.49 ± 3.09	4.16 ± 0.36	1.06 ± 0.20	35.03 ± 8.84	120.68 ± 13.75	73.92 ± 8.89
	AA	50.00 ± 12.72	24.19 ± 6.49	87.50 ± 6.36	91.50 ± 24.74	159 ± 43.84	50.50 ± 17.67	90.20 ± 31.10	135.50 ± 0.70	3.90 ± 0.56	0.98 ± 0.17	40.50 ± 6.36	125.00 ± 7.07	75.00 ± 7.07
	*P*-value	0.661	0.904	0.337	0.018	0.235	0.055	0.619	0.218	0.206	0.782	0.609	0.670	0.858
	GG	56.18 ± 12.27	24.77 ± 4.93	98.45 ± 22.10	107.45 ± 43.05	139.73 ± 38.73	38.18 ± 6.12	80.05 ± 35.32	138.00 ± 3.16	4.00 ± 0.32	0.99 ± 0.11	35.27 ± 8.27	113.89 ± 12.69	70.56 ± 8.82
CAD	GA	56.69 ± 9.26	24.50 ± 4.36	92.78 ± 12.43	112.22 ± 61.85	144.82 ± 40.45	38.18 ± 11.12	84.41 ± 34.95	140.18 ± 2.88^#^	4.43 ± 42^#^	1.11 ± 0.20	35.60 ± 10.82	114.77 ± 11.61	70.23 ± 7.99
	AA	54.13 ± 8.09	23.30 ± 3.59	99.25 ± 18.21	102.63 ± 56.64	148.00 ± 40.63	42.25 ± 12.06	86.17 ± 30.52	138.38 ± 2.32	4.22 ± 0.37	1.14 ± 0.15	37.88 ± 10.64	120.00 ± 10.69	68.75 ± 8.34
	*P*-value	0.783	0.726	0.350	0.718	0.559	0.590	0.358	0.047	0.007	0.099	0.667	0.474	0.868
	GG	52.65 ± 9.69	25.59 ± 5.42	95.73 ± 12.02	112.19 ± 39.83	151.42 ± 48.91	41.00 ± 12.38	87.98 ± 44.25	139.12 ± 2.79	4.28 ± 0.37	1.02 ± 0.18	32.38 ± 9.58	120.58 ± 11.52	75.96 ± 8.12
Ctrl	GA	55.93 ± 10.59	25.02 ± 4.97	96.73 ± 16.44	101.13 ± 36.03	142.97 ± 40.78	41.20 ± 7.35	76.50 ± 37.52	139.39 ± 3.20	4.34 ± 0.48	1.03 ± 0.22	33.73 ± 10.64	124.83 ± 16.68	76.37 ± 8.52
	AA	50.71 ± 12.07	23.46 ± 5.02	89.86 ± 15.61	121.14 ± 38.36	166.29 ± 57.22	45.86 ± 13.33	96.20 ± 47.05	138.57 ± 3.15	4.22 ± 0.53	1.14 ± 0.38	48.00 ± 0.22	113.89 ± 10.54	71.67 ± 7.07
	*P*-value	0.230	1.308	0.802	1.415	1.228	0.269	1.455	0.683	0.256	0.828	0.069	0.096	0.269

*H-Tens* Hypertension, *CAD + H-Tens* Coronary Artery Disease and Hypertension, *CAD* Coronary Artery Disease, *Ctrl* Control, *BMI* Body mass index, *FBS* Fasting blood sugar, *TC* Total cholesterol, *TG* Triglycerides, *HDL-C* High-density lipoprotein cholesterol, *LDL-C* Low-density lipoprotein cholesterol, *Na* Sodium, *K* potassium, *Cr* Creatinine, *SBP* Systolic Blood Pressure, *DBP* Diastolic Blood Pressure

Values are presented as mean ± SD. ^#^ Significant differences GG genotype (*P* < 0.05)

## Results of multinomial regression models

Results obtained from the multinomial regression model are presented in Table 4. Multinomial regression analysis was applied by adjusting for age and sex, Cr, FBS, SBP and DBP. Recessive genetic model of CD36 rs1761667 polymorphism was significantly associated with an increased risk of CAD + H-Tens (OR = 5.677; 95% CI = 1.053–30.601; *p* = 0.043). However, the dominant inheritance model ((GA + AA) vs. (GG)) of CD36 rs1761667 demonstrated a protective effect among H-Tens and CAD patients (OR = 0.170; 95% CI = 0.061–0.473; *p* = 0.001, OR = 0.218; 95% CI = 0.085–0.557; *p* = 0.001).

**Table 4:** Association between CD36 rs1761667 genotypes and the risk of CAD and hypertension with multinomial regression

Mode of inheritance	H-Tens vs. Ctrl		CAD + H-Tens vs. Ctrl		CAD vs. Ctrl	
	*P-*value	Adjusted OR [95% CI]	*P-*value	Adjusted OR [95% CI]	*P-*value	Adjusted OR [95% CI]
Autosomal recessive						
(AA) vs. (GG + GA)	0.509	1.526 [0.436–5.347]	0.043*	5.677 [1.053–30.601]	0.342	1.793 [0.538–5.979]
Autosomal dominant						
(GA + AA) vs. (GG)	0.001*	0.170 [0.061–0.473]	0.097	0.502 [0.223–1.132]	0.001*	0.218 [0.085–0.557]

*H-Tens* Hypertension, *CAD + H-Tens* Coronary Artery Disease and Hypertension, *CAD* Coronary Artery Disease, *Ctrl* Control

Adjusted for age, sex, Cr, FBS, SBP and DBP using the multinomial regression model. OR, odds ratio; CI, 95% confidence interval for the OR

*Significant differences (*p* < 0.05)

According to power calculations, the sample size provided a 94% power (*p* = 0.05).

## Discussion

Different studies on CD36 have shown that this receptor plays a key role in a number of disorders such as CVD, hypertension, and metabolic syndrome [8]. Many studies have been conducted on CD36 rs1761667 in CVD [12, 13, 16, 21], T2DM [22], metabolic syndrome [23] and obese groups [24, 25]. These studies have been carried out on different populations and in different parts of the world and the results are conflicting and the studies were small (Table 5). To the best of the authors’ knowledge, no research has been performed on subjects with CAD and hypertension on an Iranian population. Therefore, the present study assessed the association of CD36 rs1761667 gene polymorphism with susceptibility to CAD and/ or hypertension on an Iranian southeastern population. Findings revealed that the A allele might be associated with hypertension and CAD.

**Table 5:** CD36 rs1761667-related studies

Ethnicity of the population	Sample sizes		Clinical condition	minor allele	MAF	*P*-value	association results	*P*- valuae	Ref.
	case	control							
Mexican	309	132	overweight and obese	G	0.4	0.06	high serum cholesterol levels were associated with the AA genotype	0.005	[24]
Indian	250	150	T2DM	A	0.36	0.24	A allele is significantly associated with T2DM	< 0.005	[22]
Egyptian	100	100	MetS	G	0.25	0.00	Subjects with AG and GG genotypes had significantly higher systolic blood pressure, wider waist circumstance, and higher degree of dyslipidemia	< 0.001	[23]
Algerian	57	59	Obese and lean children	A	0.47	0.036	A allele was higher in obese subjects	0.03	[25]
Chongqing Han population of China	112	129	CHD	A	0.31	0.480	Significantly higher frequency of the AG genotype in the CHD group compared to the control group. The plasma levels of ox-LDL in CHD patients with AG genotype were significantly higher	0.011 0.010	[13]
Chinese Han	374	1013	atherothrombotic stroke	A	0.35	0.113	A allele were associated with atherothrombotic stroke in Chinese Han	0.020	[21]
Egyptian	71	76	CAD	A	0.46	0.680	The AG genotype involved in CAD pathogenesis as well as increased BMI, T2DM and MetS	0.001	[12]
Chinese Han	215	252	carotid atherosclerotic	A	0.33	0.135	GA genotypes increase the susceptibility of females to carotid atherosclerosis	0.041	[16]

*MAF* Minor allele frequency, *CAD* Coronary artery disease, *CHD* Coronary artery heart disease, *T2DM* Type 2 diabetes mellitus, *MetS* Metabolic syndrome, *HDL* High-density lipoprotein, *LDL* Low-density lipoprotein, *TG* Triglycerides, *BMI* Body mass index, *ox-LDL* Oxidized LDL

The crucial role that CD36 plays in hypertension has been revealed by molecular studies [15]. Expressed in renal cells such as capillary endothelium, CD36 activates the endothelial nitric oxide synthase (eNOS) via fatty acids, thereby contributing to production of nitric oxide (NO). Decreased NO activity in renal medulla is found to be associated with hypertension [26], suggesting that reduced CD36 in renal cells may be associated with hypertension [15]. In contrast to the present findings, the study carried out by Solakivi et al. on 736 Finnish subjects suffering from hypertension did not show any association between CD36 rs1761667 gene polymorphism and hypertension [27]. However, the current results showed an increased risk of hypertension in recessive inheritance model, whereas A allele carriers of rs1761667 SNP demonstrated a protective effect on hypertension. The difference between the results obtained in the present study and those of the former ones was most likely due to the racial-ethnic discrepancies and several other differences such as sample size.

Numerous studies have emphasized the role of CD36 in macrophage as a mediator of taking-up ox-LDL, so it has a special role in developing atherosclerotic plaque and CAD [28]. A previous study by Banerjee et al. evaluated the CD36 rs1761667 gene polymorphism, showing that this SNP is associated with T2DM [22]. Boghdady et al. showed that the AG genotype of the CD36 rs1761667 SNP may play a part in CAD pathogenesis and increase BMI, T2DM and metabolic syndrome in the Egyptian Sohag population [12]. Moreover, Zhang et al. obtained similar results in a study on patients with CHD from a Chinese population [13]. Contrary to the previous studies, our CAD subjects as well as those with a combination of CAD and hypertension showed a lower risk in A allele carriers of CD36 rs1761667 SNP. The reason for the differences reported in these findings as well as other studies may be the interactions of rs1761667 polymorphism with other variants in CD36 gene, ethnicity of the population, patients’ gender, sample size, differences in the genetic constitution and non-genetic or environmental properties, genetic heterogeneity, gene-environment and gene-gene interactions in various populations.

The present results showed that the A allele frequency of CD36 rs1761667 gene polymorphism was 0.37% in the Ctrl group which was similar to a study on healthy Egyptian subjects (0.46%) [12] and healthy Chinese ones (0.32%) [13]. It can be noted that diversity of ethnicity does not interfere the distribution of A allele CD36 frequency.

It has been demonstrated that CD36 binds to fatty acids and is involved in the lipid metabolism [29]. Genotypes of rs1761667 as well as the traditional risk factors of CAD such as age, sex, BMI and lipid profile were all taken into account. According to previous investigations, regarding TG, no significant difference was found between the CHD and CAD patients with different rs1761667 genotypes [12, 13]. Likewise, another study conducted by Ramos-Arellano et al. demonstrated that in a group of 232 Mexican youths with normal-weight, there was no significant difference between levels of TC, TG, and LDL-C in different genotypes of the CD36 rs1761667 polymorphism [30]. However, Bayoumy et al. revealed that subjects with metabolic syndrome had a significantly higher degree of dyslipidemia in GG and AG genotypes of CD36 rs1761667 than patients with AA genotype [23]. The only significant increase was observed in TG in the CAD + H-Tens group in GG genotype. It is hypothesized that the variety of medications used and duration of the drugs’ consumption may be the possible causes of these discrepancies.

It is noteworthy that even though the role of CD36 in multiple diseases has been revealed, it is the first study which sought to examine the genetic variation of CD36 in association with CAD and/or hypertension susceptibility.

A number of limitations should be considered when interpreting the current results which included the fact that it was difficult and time-consuming to select the H-Tens group, and hypertensive subjects without any critical coronary vessel stenosis. That is, one of the significant restrictions of the study included selection of non-CAD hypertensive patients as all the CAD hypertensive ones were subjected to coronary angiography, thus presenting a challenging and difficult task to choose hypertensive patients without any critical coronary vessel stenosis. Therefore, the sample size in this study is small. Moreover, the population of the study was restricted to southeastern Iran and, additionally, the study was a hospital-based observational study, as a result of which the findings could not be generalized to other racial/ethnic groups in other parts of the globe. Although diastolic blood pressure was higher in the control group compared to the hypertensive group, this difference was not statistically significant. Furthermore, the findings were adjusted using multinomial regression model to account for cofounders and no correction for multiple testing was done, moreover, only control genotypes were in HWE and one SNP was studied. In order to eliminate the effects of confounders such as age and sex, as well as adjusting for the baseline values of the desired variables (FBS, Cr, blood pressure, and etc.), these variables were included in regression model.

## Conclusion

The present study presented the first evidence which suggests that CD36 rs1761667 gene polymorphism might be a factor predisposing to hypertension and CAD in a southeastern Iranian population. It should be noted that the results need confirmation in further, more conclusive, and prospective studies incorporating a wide range of ethnicities with a gene expression and functional assay in the future.

## Data Availability

The dataset analyzed during the current study are available from the corresponding author on reasonable request.

## Abbreviations

ACS: Acute coronary syndrome
BMI: Body mass index
CAD + H-tens: coronary artery disease and hypertension
CAD: Coronary artery disease
CCU: Coronary care unit
CHD: Coronary heart disease
Cr: Creatinine
Ctrl: Control
CVD: Cardiovascular disease
DBP: Diastolic blood pressure
DM: Diabetes mellitus
eNOS: Endothelial nitric oxide synthase
FA: Fatty acid
FBS: Fasting blood sugar
HDL-C: High-density lipoprotein cholesterol
H-Tens: Hypertension
HWE: Hardy-Weinberg equilibrium
ICH: Intracerebral hemorrhage
K: Potassium
LDL: Low-density lipoprotein
LDL-C: Low-density lipoprotein cholesterol
Na: Sodium
NO: Nitric oxide
ox-LDL: Oxidized LDL
PCR: Polymerase chain reaction
PCR-RFLP: Polymerase chain reaction-restriction fragment length polymorphism
ROS: Reactive oxygen species
SBP: Systolic blood pressure
SNP: Single nucleotide polymorphism
T2DM: Type 2 diabetes mellitus
TC: Total cholesterol
TG: Triglycerides
